# High-Risk foreign body ingestion in children: clinical features and endoscopic outcomes

**DOI:** 10.3389/fped.2026.1832241

**Published:** 2026-06-05

**Authors:** Ning Xue, Xuxia Wei, Junjie Xu, Liping Zhu

**Affiliations:** Department of Gastroenterology, Children’s Hospital Affiliated to Shandong University (Jinan Children’s Hospital), Jinan, Shandong, China

**Keywords:** children, high-risk foreign body ingestion, management, therapeutic endoscopy, upper gastrointestinal tract

## Abstract

**Objective:**

To evaluate clinical features and endoscopic outcomes in children with high-risk upper gastrointestinal (GI) foreign bodies, stratified by object type.

**Methods:**

We retrospectively reviewed cases of children who ingested high-risk foreign bodies with upper GI retention at a Chinese tertiary pediatric center (January 2020–December 2024). Patients were stratified by the type of ingested foreign body: sharp objects, magnetic objects, or button batteries. Demographics, symptoms, retention sites, injuries, complications, and outcomes were analyzed.

**Results:**

Among 155 children, 61 (39.4%) ingested sharp objects, 42 (27.1%) magnetic objects, and 52 (33.5%) button batteries. All underwent attempted endoscopic retrieval. Groups differed significantly in age, retention site, and ingestion-to-presentation duration (all *P* < 0.05). Button battery ingestions occurred predominantly in children aged 0–3 years; sharp objects predominantly lodged in the esophagus, while magnets and button batteries were mainly retained in the stomach. Magnetic ingestions had the longest duration (>24 h) because they were often asymptomatic. Pain was more common with sharp and magnetic objects (*P* < 0.05); vomiting and perforation rates were highest in the magnetic group (*P* < 0.05), likely because only cases involving ≥2 magnets were included. Dysphagia was more frequent with sharp objects and batteries (*P* < 0.05). Esophagogastroduodenoscopy (EGD) was first-line with a success rate of 96.1%; two sharp cases needed rigid esophagoscopy, and the magnetic group had higher surgical rates (*P* < 0.05).

**Conclusion:**

High-risk foreign body ingestion is dangerous, especially ingestion of multiple magnetic foreign bodies, which are associated with high perforation and surgical rates. EGD is effective first-line therapy. Urgent intervention is needed for multiple magnets (even asymptomatic) and delayed cases. Post-removal follow-up is important, and our comparative analysis guides risk-stratified management.

## Introduction

Foreign body ingestion is a common pediatric emergency and an important public health concern. Although historical anecdotes of foreign body ingestion date back centuries, modern clinical documentation began in the mid-20th century (1). Historically, trichobezoars (hairballs) were among the most frequently reported ingested foreign bodies in children. However, with changes in household items and toy design, the spectrum of ingested objects has dramatically diversified, now including coins, toy parts, electric toothbrush heads, pins, thumbtacks, button batteries, and high-powered magnets (2). This shift has been accompanied by an increased risk of severe complications.

It is estimated that over 80,000 cases of foreign body ingestion occur annually in children in the United States alone, with the majority occurring between 6 months and 3 years of age (2). While approximately 80% of ingested foreign bodies pass spontaneously through the gastrointestinal tract without intervention, about 20% become lodged at various anatomical sites. Of these, 2%–20% require endoscopic removal, and up to 1% necessitate surgical intervention (3).

Most children remain asymptomatic and have favorable outcomes; however, a subset may develop symptoms such as pain, dysphagia, or vomiting, and are at risk for serious complications including gastrointestinal obstruction, perforation, and even death (4, 5). High-risk foreign bodies—defined as sharp objects, magnetic objects, and corrosive items (e.g., button batteries)—are particularly prone to causing mucosal injury and life-threatening complications (6).

To date, existing studies on high-risk foreign bodies are largely descriptive or based on case reports, with limited comparative data on clinical characteristics and outcomes across different types of high-risk objects (7–9). To bridge this gap, we conducted a retrospective analysis of the clinical characteristics and outcomes of pediatric patients with high-risk foreign bodies retained in the upper gastrointestinal (GI) tract, aiming to provide evidence-based guidance for their clinical management.

## Methods

### Ethics statement

This study was approved by the Ethics Committee of Shandong University Children's Hospital (approval number: SDFE-IRB/T-2026017). The requirement for informed consent was waived due to the retrospective nature of the study.

### Study design and participants

We conducted a retrospective cohort study of children aged 0–14 years who ingested high-risk foreign bodies and were managed at the Department of Gastroenterology, Shandong University Children's Hospital, between January 2020 and December 2024. Patients were classified into three groups based on foreign body type: sharp objects, magnetic objects, and button batteries. Inclusion criteria: (1) age 0–14 years; (2) clear history of foreign body ingestion; (3) confirmation of upper GI tract retention by endoscopy or imaging (e.g., x-ray, CT). Exclusion criteria: (1) imaging suggested foreign body but none found on endoscopy; (2) incomplete medical records affecting data analysis; (3) foreign body impaction secondary to pre-existing esophageal pathology (e.g., stricture, inflammation); (4) ingestion of non-high-risk objects (e.g., coins, game pieces).

### Definitions

High-risk foreign bodies included: needles, screws, irregular objects with sharp edges, date pits, animal or fish bones, magnets (specifically multiple magnets or magnet–metal combinations; single magnetic foreign bodies were excluded), and button batteries. Gastrointestinal injury was defined as mucosal injury (e.g., bleeding, ulceration) or perforation. Ingestion-to-presentation time was calculated from the time of ingestion to hospital presentation.

Diagnostic criteria for gastrointestinal perforation: Patients presented with gastrointestinal symptoms or physical signs including abdominal pain, abdominal distension, nausea, vomiting, abdominal tenderness, guarding, or rebound tenderness, plus at least one of the following: (1) imaging (X-ray, CT, or ultrasound) demonstrating subdiaphragmatic free air or other signs of perforation; (2) identification of gastrointestinal perforation on endoscopy or surgery.

### Management protocol

All patients were managed according to the Chinese Guidelines for the Management of Pediatric Gastrointestinal Foreign Bodies (2021) (6). After obtaining written informed consent for endoscopy and anesthesia, and providing detailed risk counseling (e.g., bleeding, mucosal injury), patients underwent esophagogastroduodenoscopy (EGD) under intravenous propofol sedation following a 6-hour fasting period.

Foreign bodies were retrieved using appropriate endoscopic accessories (e.g., forceps, snares, retrieval nets) selected based on object size, shape, and location. Endoscopic success was defined as complete removal of the foreign body; partial retrieval was not regarded as successful endoscopic management. For patients with mucosal injury, adjunctive interventions included hemostasis, endoscopic clipping for perforation or deep ulcers, placement of a nasojejunal tube for enteral nutrition, and antimicrobial therapy as needed. Patients with failed endoscopic retrieval were referred for surgical intervention.

### Statistical analysis

Data were analyzed using SPSS version 23.0 (IBM Corp., Armonk, NY, USA). Normality of continuous data was evaluated by the Kolmogorov–Smirnov test, and Levene's test was used to assess homogeneity of variance. Continuous variables with normal distribution and homogeneous variance were expressed as mean ± standard deviation (mean ± SD); for those with non-normal distribution or heterogeneous variance, non-parametric Kruskal–Wallis *H*-test was performed for intergroup comparisons, followed by Bonferroni-adjusted *post hoc* pairwise comparisons. Categorical variables were expressed as *n* (%) and compared using the chi-square (*χ*^2^) test. Bonferroni correction was applied for *post hoc* multiple comparisons of categorical variables. A two-sided *P* < 0.05 was considered statistically significant.

## Results

### Baseline characteristics of patients

**Figure 1:**
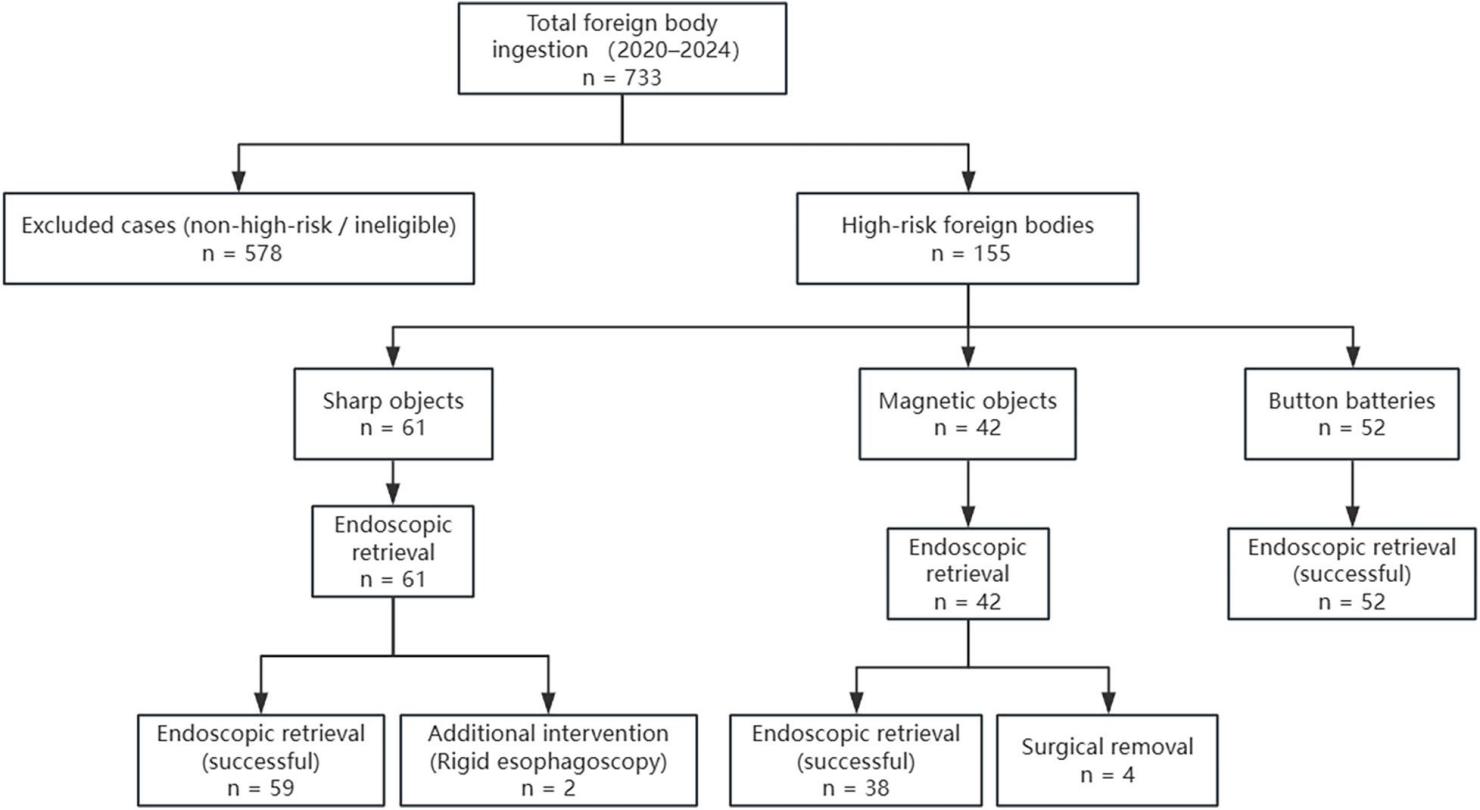
Flowchart of patient enrollment and management outcomes. A total of 733 children with foreign body ingestion (2020–2024) were screened. Of these, 578 cases were excluded due to non-high-risk foreign bodies or ineligible criteria. The remaining 155 high-risk cases were stratified into sharp objects (*n* = 61), magnetic objects (*n* = 42), and button batteries (*n* = 52). Management outcomes included successful endoscopic retrieval, additional intervention (rigid esophagoscopy), or surgical removal.

**Figure 2:**
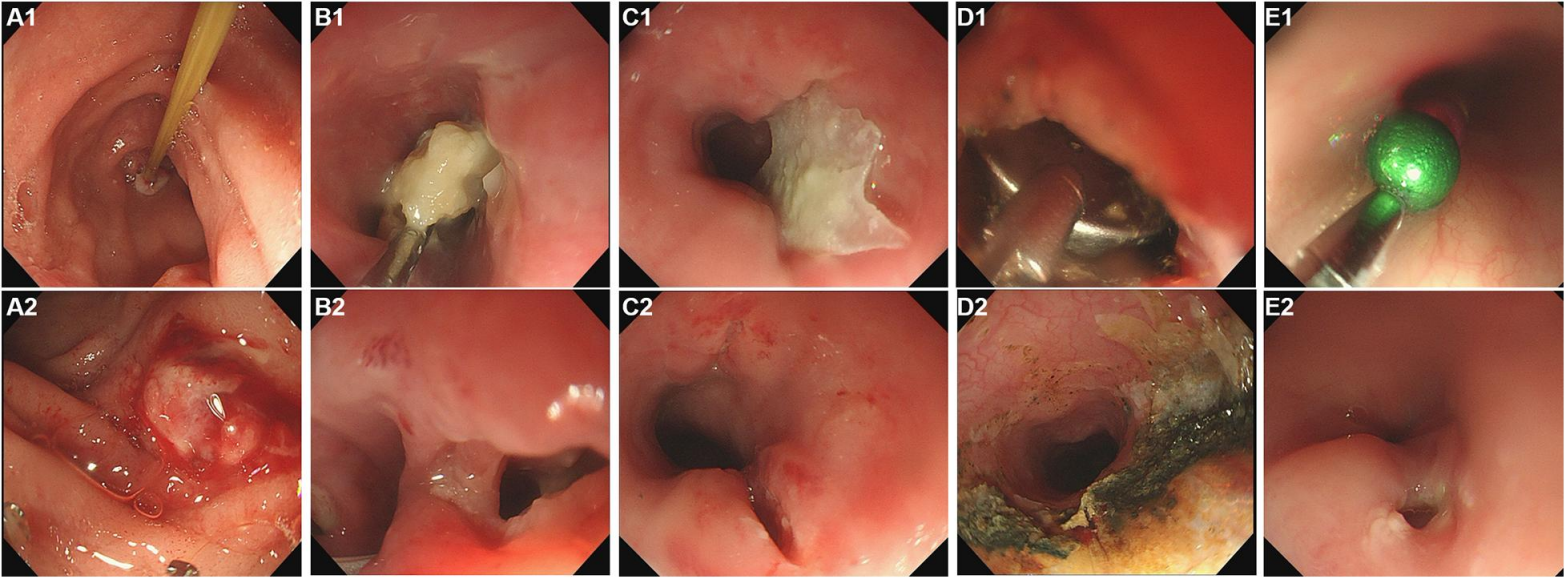
Endoscopic findings of various foreign bodies in pediatric patients. **(A)** A toothpick retained in the descending duodenum causing mucosal injury; **(B)** A date pit lodged in the esophagus leading to perforation; **(C)** A fish bone impaction in the esophagus resulting in perforation; **(D)** A button battery retained in the esophagus causing mucosal erosion and chemical injury; **(E)** Magnetic beads lodged at the gastroesophageal junction and gastric fundus causing perforation.

The patient enrollment and management process is illustrated in Figure 1. A total of 155 children were included in the study, of whom 95 (61.29%) were male and 60 (38.71%) were female, with a mean age of 4.07 ± 3.24 years. The cohort was divided into three groups based on foreign body type: 61 patients (39.35%) in the sharp objects group [32 males [52.46%], 29 females [47.54%]; mean age 5.47 ± 4.05 years], 42 patients (27.10%) in the magnetic objects group [27 males [64.29%], 15 females [35.71%]; mean age 4.19 ± 2.39 years], and 52 patients (33.55%) in the button battery group [36 males [69.23%], 16 females [30.77%]; mean age 2.35 ± 1.54 years]. Sharp objects included needles, screws, toothpicks, date pits, animal or fish bones, and badge-like items with sharp edges (Figures 2A–C); battery types included button and cylindrical batteries (Figure 2D); and magnetic objects consisted primarily of magnet spheres and blocks (Figure 2E). During the same period, our center managed a total of 733 pediatric foreign body ingestions.

There was no significant difference in sex distribution among the three groups (*P* > 0.05). However, the mean age of children in the button battery group was significantly lower than that of children in both the sharp object group (*P* = 0.000) and magnetic object group (*P* = 0.001). Significant intergroup differences were observed in age distribution, retention site, and mean ingestion-to-presentation time (all *P* < 0.05). Specifically, the majority of button battery ingestions occurred in children aged 0–3 years; sharp objects were predominantly retained in the esophagus, whereas magnetic and button battery objects were mainly located in the stomach; and ingestion-to-presentation time was longest in the magnetic object group, with most cases exceeding 24 h (Table 1).

**Table 1:** Patient demographics and clinical characteristics.

Basic Characteristics	Sharp Object Group (*n* = 61)	Magnetic Object Group (*n* = 42)	Battery Object Group (*n* = 52)	*H*/*χ*^2^	*P*
Mean age (years)	5.47 ± 4.05	4.19 ± 2.39	2.35 ± 1.54 ^a^^,^^b^	23.418	0.000
Age distribution (%)					
0–3years	27 (44.27%)	18 (42.86%)	40 (76.93%)	39.596	0.000
4–6 years	8 (13.11%)	18 (42.86%)	11 (21.15%)		
7–14 years	26 (42.62%)	6 (14.28%)	1 (1.92%)		
Sex (%)					
Male	32 (52.46%)	27 (64.29%)	36 (69.23%)	3.546	0.170
Female	29 (47.54%)	15 (35.71%)	16 (30.77%)		
Retention site (%)					
Esophagus	33 (54.1%)	0 (0%)	13 (25%)	76.522	0.000
Stomach	18 (29.5%)	30 (71.42%)	37 (71.15%)		
Duodenum/jejunum	10 (16.39%)	3 (7.14%)	2 (3.85%)		
Multiple sites	0 (0%)	9 (21.44%)	0 (0%)		
Ingestion-to-presentation time (%)					
≤24h	53 (86.89%)	20 (47.62%)	45 (86.54%)	25.77	0.000
>24h	8 (13.11%)	22 (52.38%)	7 (13.46%)		

a Compared with the sharp object group, *P* < 0.05.

b Compared with the magnetic object group, *P* < 0.05.

### Clinical manifestations and complications

Following ingestion, some children developed symptoms including pain (chest or abdominal), dysphagia, vomiting, cough, and fever. The sharp and magnetic object groups had significantly higher rates of pain compared to the button battery group (*P* < 0.05). The magnetic object group exhibited significantly higher rates of vomiting and perforation than both the sharp and button battery groups (*P* < 0.05). In contrast, dysphagia was more frequent in the sharp and button battery groups than in the magnetic group (*P* < 0.05) (Table 2).

**Table 2:** Clinical manifestations and gastrointestinal injuries in children with ingested foreign bodies.

Clinical Presentation and Gastrointestinal Injury	Sharp Object Group (*n* = 61)	Magnetic Object Group (*n* = 42)	Battery Object Group (*n* = 52)	*χ* ^2^	*P*
Pain (%)	30 (49.18%)	20 (47.62%)	12 (23.08%)^a^^,^^b^	9.363	0.009
Vomiting (%)	11 (18.03%)	18 (42.86%)^a^	10 (19.23%)^b^	10.875	0.004
Dysphagia (%)	19 (31.15%)	0 (0%)^a^	10 (19.23%)^b^	15.881	0.000
Coughing (%)	1 (1.64%)	0 (0%)	4 (7.69%)	4.010	0.086
Fever (%)	0 (0%)	3 (7.14%)	2 (3.84%)	5.608	0.061
Mucosal injury (%)	23 (37.7%)	23 (54.76%)	25 (48.07%)	3.078	0.215
Perforation (%)	3 (4.91%)	18 (42.86%)^a^	1 (1.92%)^b^	39.071	0.000

a Compared with the sharp object group, *P* < 0.05.

b Compared with the magnetic object group, *P* < 0.05.

### Treatment and outcomes

Endoscopic retrieval via EGD was successful in 149 patients (96.13%). Four patients (2.58%) in the magnetic object group underwent partial retrieval due to multi-site retention, and two patients (1.29%) with esophageal foreign bodies failed EGD and required rigid esophagoscopy for removal. The rate of surgical or alternative intervention was significantly higher in the magnetic object group compared to the sharp and button battery groups (*P* < 0.05) (Table 3).

**Table 3:** Treatment modalities and clinical outcomes in children with ingested foreign bodies.

Treatment and Outcome	Sharp Object Group (*n* = 61)	Magnetic Object Group (*n* = 42)	Battery Object Group (*n* = 52)	*χ* ^2^	*P*
Endoscopic retrieval (%)	59 (96.72%)	38 (90.48%)	52 (100%)	5.758	0.056
Surgical removal (%)	0 (0%)	4 (9.52%)^a^	0 (0%)^b^	10.736	0.004
Stricture formation (%)	0 (0%)	0 (0%)	2 (3.85%)	4.421	0.110
Mortality (%)	0 (0%)	0 (0%)	0 (0%)	0.000	1.000

a Compared with the sharp object group, *P* < 0.05.

b Compared with the magnetic object group, *P* < 0.05.

## Discussion

Foreign body ingestion is a prevalent pediatric emergency, and high-risk objects (sharp, magnetic, button batteries) are known to be associated with severe complications, as documented in numerous previous studies (10–13). While prior research has advanced our understanding of single-type high-risk foreign bodies and their management, targeted comparative data among these three categories remains scarce—specifically regarding differences in age distribution, complication profiles, and treatment strategies. For instance, button batteries are most commonly ingested by toddlers, sharp objects are prone to impaction in the esophagus, and magnetic foreign bodies are associated with the highest surgical intervention rate. Building on this existing foundation, this study conducted a comprehensive comparative analysis of these three high-risk upper gastrointestinal foreign bodies to supplement current knowledge and provide actionable evidence for optimizing risk-stratified management.

As sharp objects are prone to impaction in the esophagus, they were predominantly lodged in this site, while magnetic and button battery objects more frequently reached the stomach (14–16). This anatomical distribution directly determines the differences in treatment selection: rigid esophagoscopy was required in only two cases of esophageal sharp objects after the failure of flexible endoscopy, highlighting the utility of rigid instruments for tightly impacted proximal esophageal foreign bodies, whereas no cases in the other two groups required rigid esophagoscopy.

Notably, the perforation rate of magnetic foreign bodies in our study was higher than that reported in previous literature. The primary reason is that earlier studies included ingestions of single magnetic objects in their analyses, whereas our study exclusively enrolled cases involving two or more magnets or magnet–metal combinations. This approach corrects the bias introduced by “mixed statistics” in prior reports and provides a more accurate risk assessment for truly high-risk magnetic ingestions. Given the high perforation and surgical intervention rates for multiple magnetic foreign bodies, consistent with clinical guidelines, we endorse urgent endoscopic retrieval within 2 h for confirmed upper GI retention of multiple magnets or magnet–metal combinations, even in asymptomatic patients (17–20). A key finding supporting this urgency is that the magnetic object group had the longest mean ingestion-to-presentation interval (most >24 h), attributable to their insidious and often asymptomatic course—symptoms typically manifest only after complications (bowel wall compression, necrosis, perforation) due to multiple magnets trapping adjacent bowel loops (21–24). Our data, which demonstrated high perforation and tissue injury risks in such delayed cases, further supports prioritizing prompt endoscopic removal over prolonged observation. Enhanced detection (e.g., ultrasound) and frequent monitoring should be reserved for cases where immediate retrieval is not feasible, serving as adjunctive measures to monitor potential complications before definitive intervention.

EGD-assisted retrieval was the first-line therapy for all three groups, with a 96.13% success rate (25–27). Notably, our data showed a high incidence of mucosal injury across all three groups even after active endoscopic treatment, underscoring the importance of post-removal follow-up management to mitigate potential delayed complications.

We acknowledge study limitations: the analysis was largely univariate and descriptive, lacking multivariable analysis to identify independent predictors of adverse outcomes; defining ingestion-to-presentation time introduced potential recall bias; and the single-center, retrospective design with limited long-term follow-up failed to fully capture delayed complications (e.g., esophageal stricture post-button battery ingestion).

In summary, this study provides valuable comparative data on three high-risk pediatric upper GI foreign bodies. Key findings include: confirmation of rigid esophagoscopy utility for tightly impacted sharp objects; a refined perforation risk estimate for multiple magnetic ingestions through exclusion of low-risk single-magnet cases; the need for active evaluation of delayed magnetic foreign body presentation; and quantification of the disproportionate intervention burden associated with magnetic ingestions. Collectively, these findings offer actionable guidance to optimize risk-stratified management and reduce delayed complications. Future prospective, multicenter studies incorporating multivariable analysis and long-term follow-up are needed to validate our observations.

## Data Availability

The raw data supporting the conclusions of this article will be made available by the authors, without undue reservation.
